# Selection of *Beauveria* isolates pathogenic to adults of *Nilaparvata lugens*

**DOI:** 10.1093/jis/14.1.32

**Published:** 2014-01-01

**Authors:** Maoye Li, Shiguang Li, Amei Xu, Huafeng Lin, Dexin Chen, Hui Wang

**Affiliations:** 1 School of Plant Protection, Anhui Agricultural University, Hefei 230036, Anhui Province, China; 2 Qingzhou Tobacco Research Institute, China National Tobacco Corporation, Qingdao 266001, Shandong Province, China; 3 These authors contributed equally to this work

**Keywords:** biological characteristics, chitinase, microbial control, virulence

## Abstract

The brown planthopper, *Nilaparvata lugens* Stål (Hemiptera: Delphacidae), is a destructive invasive pest and has become one of the most economically-important rice pests in China. Effective control measures are desperately needed. Entomopathogenic fungi, such as *Beauveria bassiana* (Balsamo-Crivelli) Vuillemin (Hypocreales: Clavicipitaceae) and *B. brongniartii* (Saccardo), have shown great potential for the management of some sucking pest species. In this study, to explore alternative strategies for sustainable control of the sucking pest population, nine isolates of *Beauveria* from different pests were bioassayed under the concentrated standard spray of 1000 conidia/mm ^2^ in laboratory. The cumulative mortalities of adults ranged from 17.2 to 79.1% 10 days after inoculation. The virulence among all tested isolates exhibited significant differences (at *p* = 0.05). The highest virulent isolate was Bb09, which killed 79.1% of the treated insects and had a median lethal time of 5.5 days. Its median lethal concentration values were estimated as 134 conidia/mm ^2^ on day 10. The chitinase activities of nine isolates were also assayed. The results showed that the chitinase activity (18.7 U/mg) of isolate Bbr09 was the highest among all tested isolates. The biological characteristics of these strains, including growth rate, sporulation, and germination rate, were further investigated. The results showed that strain Bbr09 exhibited the best biological characteristics with relatively higher hyphal growth rate, the highest spore production, and the fastest spore germination. The isolate of Bbr09 had strong pathogenicity and exhibited great potential for sustainable control of *N* . *lugens* .

## Introduction

The brown planthopper, *Nilaparvata lugens* Stål (Hemiptera: Delphacidae), is one of the major rice pests throughout Asia. At present, chemical insecticides are still the only means utilized for the control of *N. lugens* . However the consequential problems of chemical insec-tides are of significant environmental concern. The extensive use of chemical insecticides can cause target insect resistance, detrimental impact on the natural enemies, health and environmental hazards, residue persistence, and development of tolerance ( Liu et al. 2003 ; Jin et al. 2008 ; Kontsedalov et al. 2012 ). Therefore, there is a need for alternatives, and biocontrol may be an effective alternative to chemical control while also being friendly to environment ( Faria and Wraight 2001 ).

Entomopathogenic fungi are important worldwide biological control agents, and a number of them are already commercially available ( Peveling and Demba 1997 ; Faria and Wraight 2007 ; Rodriguez et al. 2009 ). At present, *Beauveria bassiana* (Balsamo-Crivelli) Vuillemin (Hypocreales: Clavicipitaceae), *Metarhizium anisopliae* (Metachnikoff) Sorokin, and *Metarhizium flavoviride* Gams and Rozsypal, which are studied intensively, have the potential to be used for biological control of sucking pests, such as aphids (Vandenberg et al *.* 2001; Shan and Feng 2010 ), whiteflies ( Abdel-Raheem et al. 2009 ; Cuthbertson et al *.* 2011), leafhoppers ( Feng et al. 2004 ; Pu et al. 2005 ), and planthoppers (Toledo et al *.* 2007, 2008; Kiran and Veeranna 2012 ). It was reported that these entomopathogenic fungi appeared to be the most efficient because of their ease of virulence, massive production, storage, and application. As for fungal insecticide development, virulence of fungal biological control agents is usually used as a primary criterion for isolate selection in addition to other biological characteris-characteristics ( Samuels et al. 1989 ; Reay et al. 2008 ; Petlamul and Prasertsan 2012 ). Virulence is the most important indicator when measuring the potential of fungi against a pest and is the basis for choosing highly virulent fungi in laboratory bioassays ( Roberts and Leger 2004 ; Jin et al. 2008 ). Entomopathogenic fungi cause infection by active penetration through the cuticle of insects and release extracellular cuticle-hydrolyzing enzymes that participate in the decomposition of protein, chitin, and lipids, which are the principal components of the cuticle ( St. Leger et al. 1986 ; Pedrini et al. 2007 ; Schrank and Vainstein 2010 ). Different studies suggest that chitinases and proteases are major determinants of fungal virulence in the complex and multifactorial insect pathogen relationship ( Fan et al. 2007 ; Pelizza et al. 2012 ). Therefore, more and more laboratory bioassays continue to be conducted in order to obtain a fungal isolate with higher efficacy for integrated *N. lugens* control.

In this study, nine strains of *Beauveria* collected from different pest species were selected, and their virulence on *N. lugens* adults and biological characteristics, including colony morphology and growth rate, conidial germination, and chitinase activity, were assessed for the selection as a suitable biological control agent candidate in the management of *N. lugens* .

## Materials and Methods

### Plant culture

*Oryza sativa* L. (Poales: Poaceae) cv. Nongfengyou, a rice cultivar susceptible to *N. lugens* , was prepared: 10-day-old rice seedlings in dishes (15 cm diameter) were transplanted in plastic pots (20 cm diameter, 20 cm high), which were filled with nutrition solution and cultured for another 10 days under controlled conditions (25–30°C; 14:10 L:D photoperiod; 80% RH) in the greenhouse at the School of Plant Protection, Anhui Agricultural University, Hefei, China.

### *N. lugens* population rearing

**Figure 1.**
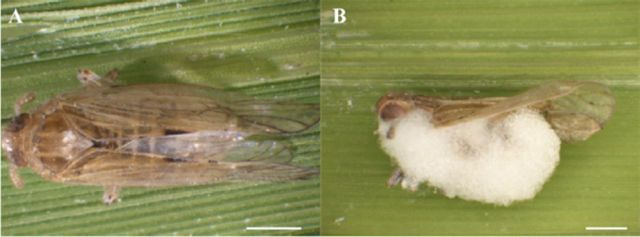
(A) Healthy adult *Nilaparvata lugens* . (B) Adult *N.lugens* (96 hr) after death caused by *Beauveria* (isolate Bbr09). Bar: 500 μm. High quality figures are available online.

Adult *N. lugens* ( Figure 1 A), originally collected from the rice fields in Anhui Agricultural University (No. 130 West Changjiang Rd, Hefei, Anhui, China), were reared on the rice seedlings described above. The condition stimulated the copula and oviposition by females. Nymphs that were born from these ovipositions and grew up to be adults were used in pathogenicity assays.

### Sources of fungi

The fungal isolates of *B. bassiana* and *B. brongniartii* (Saccardo) used in the experiment were provided by Biocontrol Reserch Lab, Anhui Agricultural University, Hefei, China, and originally isolated from a broad spectrum of insects ( Table 1 ). In order to confirm virulence of the isolates was not attenuated, original host insects were inoculated with these isolates and the fungi were re-isolated on Sabouraud dextrose agar yeast medium. The conidia colonies were inoculated to Sabouraud dextrose agar yeast medium in Petri dishes (9 cm diameter) and maintained at 25 ± 1°C in darkness for 15 days. After being scraped from the plate, conidia were dried to a water content of 5% at ambient temperature with a vacuum drier (VirTis, SP Scientific, www.spscientific.com ). Dry conidia were preserved at 4°C in darkness for use as soon as possible in all tests, warranting ≥90% viability.

**Table 1.**
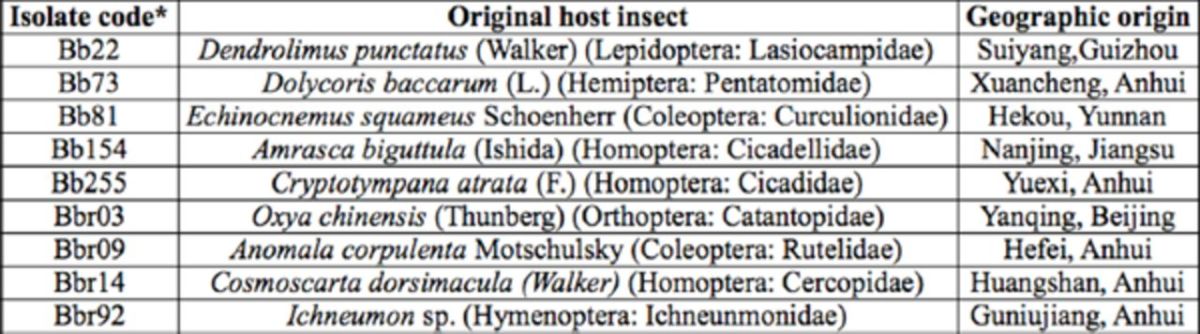
List of fungal species screened against adults of *Nilaparvata lugens* , original host, and geographic origin. Isolate codes follow fungal number of research center of entomopathogenic fungi.

**Table 2.**
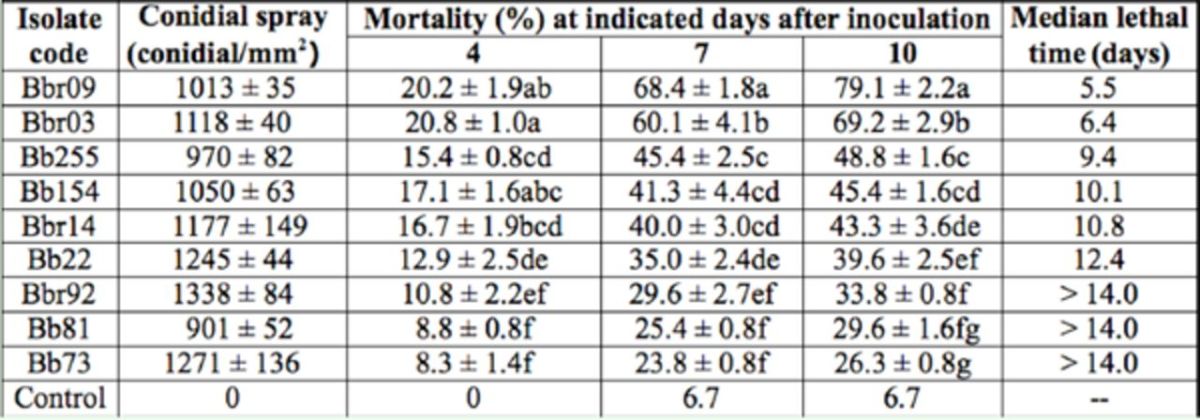
Bioassay results for adults of *Nilaparvata lugens* representing isolates of *Beauveria.*

Mean ± SD was estimated from three replicate bioassays. Means with the same letter in the same column are not significantly different ( *p* > 0.05) by ANOVA followed by Tukey’s test. CK = control.

Conidia of each fungal isolate were transferred into corresponding test tubes (3 cm diameter, 20 cm length) and suspended in sterile distilled water by shaking in 10 mL flasks containing 60 to 72 glass beads (3 mm diameter). No surfactant [0.02% Tween-80 (polyoxyethylene sorbitan monolaurate)] was added, since 5 min of agitation at 700 oscilla-tions on a mechanical shaker homogenize suspensions of viable single conidia. The suspensions were then adjusted to 1 ×10 ^8^ conidia/mL by appropriate dilution based on hemocytometer counts.

### Bioassays with *N. lugens* adults

Two different series of bioassays were conducted. For each fungal isolate, 60 adults (approximately 10 days old) from the rice seedlings in the greenhouse were aspirated into test tubes (3 cm diameter, 20 cm length). Afterwards, they were transferred into a transparent bell glass jar (6 cm diameter, 20 cm high) with a bottle cap that had small holes for air circulation to avoid adults escaping. The suspensions of 1 ×10 ^8^ conidia/mL in sterile water with 0.05% (v/v) Tween-80 that was prepared previously were sprayed onto adults and rice seedlings in the glass jar by a hand-held Micro Ultra sprayer (Micron Sprayers, www.micron.co.uk ). The concentration of conidia deposited onto the adults and seedlings was measured as number of conidia/mm ^2^ using microscopic (400 ×magnification) counts of conidia collected onto four glass slips (20 ×20 mm) under each spray. Four replicates per isolate were used, and a blank control was sprayed with 0.05% (v/v) Tween-80 for each fungal isolate. All treatments were maintained at 25 ± 1°C, a 14:10 L:D photoperiod, and 80% RH, with adult mortality recorded daily for 10 days posttreatment. The dead adults were transferred every day into 70% ethanol for 10 sec, washed in sterile distilled water, then treated with 0.5% sodium hypochlorite for 30 sec, and washed again in sterile distilled water. After that, the specimens were placed in Petri dishes (9 cm diameter) with filter paper that was moistened with sterile distilled water and maintained in a humidity chamber (saturated atmosphere) for fungal sporulation at 25 ± 1°C for three to five days.

The second series of bioassays included only those isolates that caused greater than 50% adult mortality in the first series of experiment. Bioassays used aqueous *B* . *brongniartii* (Bbr03 and Bbr09) suspensions (1 ×10 ^6^ , 1 × 10 ^7^ , and 1 × 10 ^8^ conidia/mL) containing 0.02% Tween-80 in distilled water (the control). The bioassays were repeated four times using the same method described above.

### Fungal characteristics, colony growth rates, and conidial yields

The isolates were cultured on Sabouraud dextrose agar yeast medium in Petri dishes (90 mm diameter), with four replicates for each isolate. The dishes were maintained in an incubation chamber at 25 ± 1°C and RH ≥80% in the dark. The diameters of the colonies were measured five, 10, and 15 days after incubation.

After 15 days, a culture disc from the center of each fungal colony was removed using a sterile 13 mm diameter punch and placed into a 50 mL flask with 20 mL sterile 0.05% Tween-80. Each isolate contained four flask cultures as replicates. The conidial suspensions of the nine isolates were prepared by stirring as described above. Conidial concentrations were determined using a hemocytometer according to the method by Zhang et al. (2011) .

### Conidial germination

Fifteen µL of each conidial suspension were transferred to a sterile glass slide smeared with Sabouraud dextrose agar yeast. The slide was incubated in at 25 ± 1°C and RH > 90%. Four replicates were set up for each isolate. Conidial germination was estimated at 24 hr by counting the germinated and non-germinated conidia in a random field of vision sample on each slide using a microscope at 400×magnification. The number of germinated and non-germinated conidia from four microscopic view fields (100 conidia/field) was counted. For each observation, 400 conidia were examined in each replicate slide. The mean percent germination was calculated for each sample.

### Chitinase activity assay

The enzymatic reaction was initiated by addition of 200 µL isolated chitinase solution to the colloidal chitin (10% w/v chitin in 300 µL acetate buffer, pH 5.0) as substrate and continued for 60 min at 50°C. The hydrolysis of chitin was measured by the para-dimethyl-amino benzaldehyde reagent method according to Reissig et al. (1955) .

The reaction was cooled to room temperature, centrifuged at 5,000 ×g for 10 min, and the absorbance at 540 nm (A540) was taken against water as blank. The enzyme activity was measured according to a standard curve developed using known concentrations of N-acetyl-d-glucosamine. One enzyme unit (U) was defined as the amount of enzyme that produced 1 µM of N-acetyl glucosamine per minute under the above conditions.

### Statistical analyses

The mortalities (%) of *N. lugens* in treatments (M2) were corrected by the mortality (%) in blank controls (M1) according to Abbortt's formula: Mc = (M2 -M1) / (100-M1). All corrected mortalities (%) data were subjected to an arcsine transformation prior to analysis of variance to detected differences in mortalities of *N. lugens* . Mean separation was performed using Tukey’s honestly significant difference test ( *p* < 0.05). Statistical analyses were performed using the DPS program ( Tang and Feng 2007 ).

## Results

### Infection of fungal pathogens to *N. lugens* adults

It was found that *N. lugens* adults infected with *Beauveria* moved sluggishly in the early stage of infection, female adults stopped oviposition, and nymphs had difficulty in peeling. The infected *N. lugens* adults then died holding rice stems, which were not found in the control group ( Figure 1 A). As the disease progressed, *N. lugens* adults’ bodies turned brown when infected with *Beauveria* for three days, and white flocculent hyphae grew out of the insect body from the segmaco-ria, somatic fold sag, foot joints, etc. In the following one to two days, the mycelium covered the whole body until white or light yellow conidia ( Figure 1 B) emerged. From inoculation to the completion of the entire process of intrusion infection, tissue lesions, host’s death, and in vitro sporulation, the shortest time needed was four days, and the longest nine days.

### Virulence test on *N. lugens* adults

The cumulative corrective mortalities of *N. lugens* differed significantly among the 10 isolate treatments (F8, 27 = 269.5, *p* < 0.01) on the 10th day after spraying. Only two isolates, Bbr03 and Bbr09, killed more than 50% of the adults, of which Bbr09 had the lowest LT50 of (5.5 days). Cumulative adult mortality increased with conidial concentration. Based on the estimates of the LC50 and associated 95% CI ( Table 3 ), the two isolates, Bbr09 and Bbr03, had LC50s of 134 and 443 conidia/mm ^2^ on day 10, respectively.

**Table 3.**
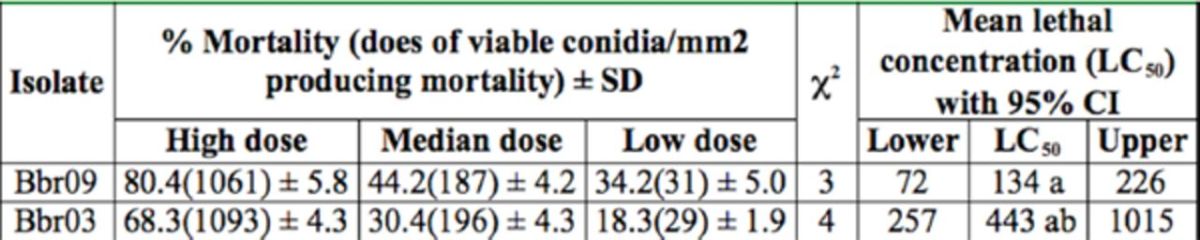
Virulence of the selected isolates Bbr03 and Bbr09 towards *Nilaparvata lugens* adults.

Mean ± SD was estimated from three replicate bioassays. Means with the same letter in the same column are not significantly different ( *p* > 0.05, by ANOVA followed by Tukey’s test.

### Fungal characteristics, colony growth rates, and conidial yields

Viewing from biological characteristics of the original 15 days of culture ( Table 4 ), colony growth rates (5 days: F8, 27 = 60.7, *p* < 0.01; 10 days: F8, 27 = 177.1, *p* < 0.01; 15 days: F8, 27 = 247.4, *p* < 0.01), conidial yields (F8, 27 = 477.7, *p* < 0.01), and spore germination (F8, 27 = 31.8, *p* < 0.01) of the nine isolates were tested, and the results differed significantly among them. The isolate Bbr09 generally had the fastest colony growth rates and the highest conidial yields. Spore germination of all tested isolates exceeded 80% within 24 hr.

**Table 4.**
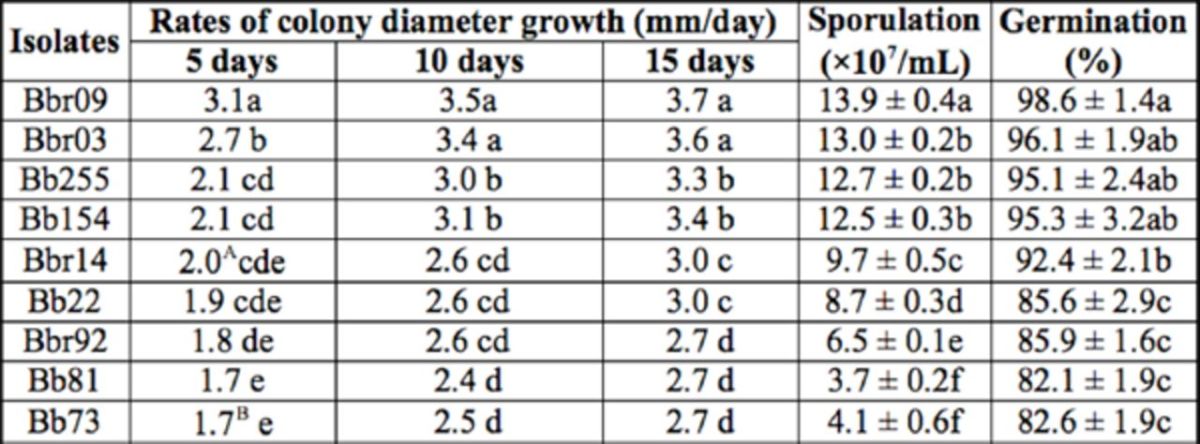
Characteristics of the tested *Beauveria bassiana* (Bb) and *B* . *brongniartii* (Bbr) isolates on medium.

Data are given in mean ± standard error (SE); values followed by the same letters do not differ significantly according to Tukey's honestly significant difference test ( *p* < 0.05). SE’s are all < 0.1 unless indicated in rates of colony diameter growth: A = 0.18, B = 0.16.

### Chitinase activities

**Figure 2.**
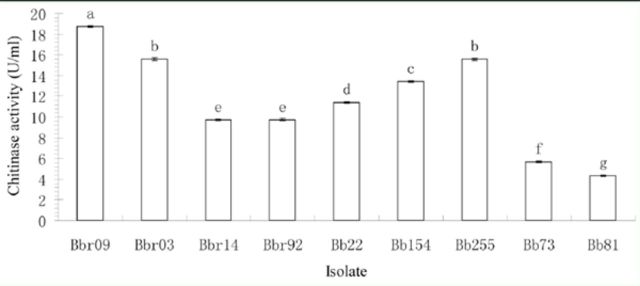
Chitinase activity assay of the examined strains. The letters on the top of columns show the significance of difference. High quality figures are available online.

The chitinase activities of nine isolates demonstrated a significant variation ( Figure 2 ; *p* < 0.01). The three strains Bbr09, Bbr03, and Bb255 had high chitinase activities, among which Bbr09 had the highest chitinase activity of (18.7 U/mL), whereas, the isolate Bb81 showed the least chitinase activity (4.3 U/mL).

## Discussion

In this research, no relationship between pathogenicity and the isolates’ original hosts was observed, even though most authors agree that isolates of entomopathogenic fungi are generally more pathogenic to the species of insect that they are isolated from or a closely related species ( Zimmermann 1982 ; Poprawski et al. 1985 ). The results of our study do not support this point of view, since the most pathogenic isolate, *B. brongniartii* Bbr09, was isolated from larvae of *Anomala corpulenta* Motschulsky (Coleoptera: Rutelidae) living in corn crops. The results from this study suggest that isolates from natural environments have the potential to be highly pathogenic, and the screening of entomopathogenic fungi should not be limited only to isolates from the target host or its close relatives.

As the results of this study showed, isolates with higher chitinase activity had relatively higher virulence. The strain Bbr09, which had the highest chitinase activity among the test isolates, also caused the highest adult mortality. Studies have shown that the chitinase secreted from *B. bassiana* ( Gupta et al. 1992 ; Havukkala et al. 1993 ; Pelizza et al. 2012 ), *M. anisopliae* ( St. Leger et al. 1991 ), *M. flavoviride* ( St. Leger et al. 1993 ), and *Nomuraea rileyi* ( El-Sayed et al. 1989 ) was closely related to strain virulence. Fang et al. (2005) showed that highly expressed chitinase ( *Bbchit1* ) of *B. bassiana* could significantly improve the insecticidal virulence, and this phenomenon has been found in both fungi and bacteria. Research on *Beauveria* and *Metarhizium* infecting *Peregrinus maidis* by Toledo et al. (2010) showed a similar phenomenon, as during the process of conidia germinating and penetrating epidermis, the secreted chitinase, esterase, and extracellular protease destroyed insect body surface and promoted the successful invasion of hyphae. During the process of entomopathogenic fungi infecting insects, chitinase not only participated in the degradation of the insect body wall by itself, but also played did so together with other enzymes, such as protease.

This research showed that biological characteristics of fungi strains were related to virulence. Zhang et al. (2011) found that the average growth speed, spore production quantity, and germination percentage of the strains were all related to virulence of the strains during the process of screening *Beauveria* , which had high virulence to *Dendroctonus valens.*Petlamul and Prasertsan (2012) argued that strains with higher germination rate had stronger virulence to *Spodoptera litura* . However, Liu et al. (2003) reported that no obvious correlation between germination rate and virulence of the fungal isolates was found in *Beauveria* and *Metarhizium* isolates against *Lymantria xylina* . In this study, colony growth rates, conidial germination rates, and spore production quantity of isolate Bbr09 were all superior to those of the other eight indoor saved strains. A shorter time needed for producing spores and a higher unit area of spore production quantity meant a higher production efficiency during the industrial production of fungal pesticides ( Feng et al. 1994 ; Kassa et al. 2008 ); meanwhile, in the natural environment, strains would produce more spores for the next infection and diffusion cycle after finishing infecting the host insect.

In this study, bioassay results of the new strain *B. brongniartii* Bbr09, isolated from the field, showed it had the highest (79.1%) accumula-tive corrective mortality and the least LT50 (5.5 days) to *N. lugens* among the test strains. The study on chitinase activity, conidium infection ability, and biological characteristics showed the isolate Bbr09 was superior to those of other strains. The isolate Bbr09 was found to be efficienct in the control of eggs of *N. lugens* ( Li et al. 2012 ). This strain had high insecticidal efficiency and was easily produced, showing that it has great application potential in field control of *N. lugens* .
